# Supplementary material for: Network motifs shape distinct functioning of Earth’s moisture recycling hubs
Source: Nat Commun. 2022 Nov 2;13:6574. doi: 10.1038/s41467-022-34229-1 (PMC9630528; doi:10.1038/s41467-022-34229-1)
Supplement: Supplementary file 1 — Supplementary Information [file 41467_2022_34229_MOESM1_ESM.pdf]

Supplementary information

Network motifs shape distinct functioning of Earth's moisture recycling hubs

N. Wunderling, F. Wolf, O.A. Tuinenburg & A. Staal

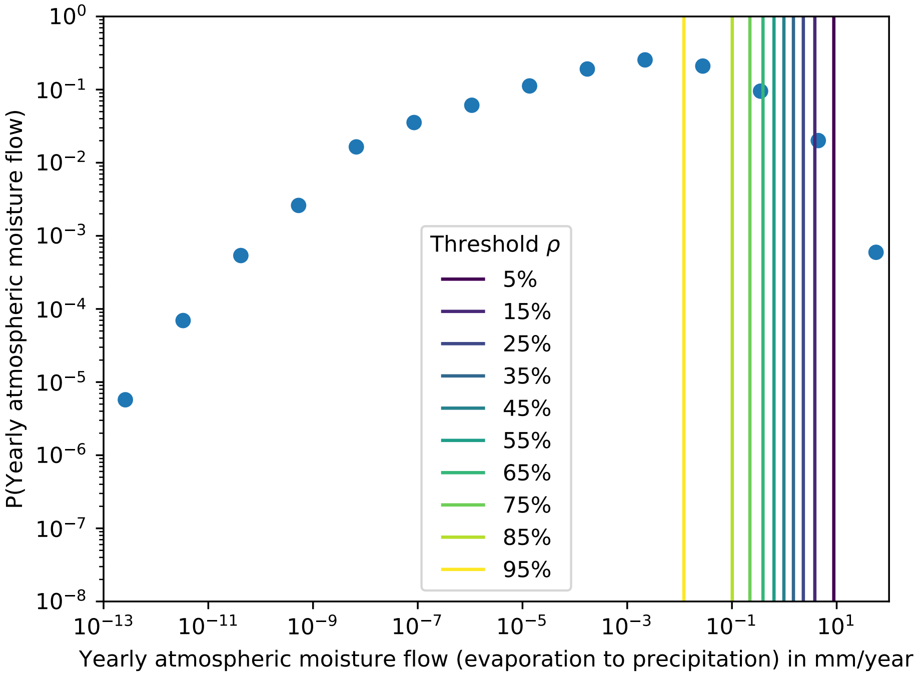

Figure S1 | Probability distribution of moisture transport volume from grid cell to grid cell. The x-axis shows the yearly atmospheric moisture transport per edge in millimeter per year. Vertical lines indicate thresholds at which the strong links (right of the vertical line) carry a certain share of the total moisture volume.

Supplementary Discussion

In- and out-degree distribution

The degree distribution reveals characteristic features of a network. For example, scale-free networks with power-law-like degree distributions include so-called hubs, nodes with orders of magnitude more links than most of the nodes of the network. In contrast, random networks exhibit a Poisson-distribution for the degree, allowing for degrees in a much narrower range of values[1]. For this reason, we analyze the in- and out-degree distribution of the global moisture recycling network.

Figs. S2 and S3 show the in- and out-degree distribution for the all-to-all network (S2) and the land-to-land network (S3). In both, the in-degree is marked with a plus (+) and the out-degree by a dot (o), whereas the color code refers to the different network thresholds ρ described in the sensitivity analysis (see SI Chapter 2). As expected, for a higher threshold ρ and, thus, a higher link density (more links in the network) the distributions get broader. Additionally, all distributions exhibit rather stable values over two orders of magnitude (from 10^0 to 10^2) before the probability drops drastically. The similarity between the different distributions for the thresholds and for the different connectivity patterns is remarkable and further confirms the qualitative robustness of the analysis against changes of the moisture recycling threshold ρ.

Comparing the in- and out-degree distributions shows that the in-degree is consistently (without exception) broader distributed than the out-degree (compare positions of the plus and the dots in Fig. S2 and Fig. S3 in the tails). Hence, the network is more characterized by super-receivers rather than by super-distributors.

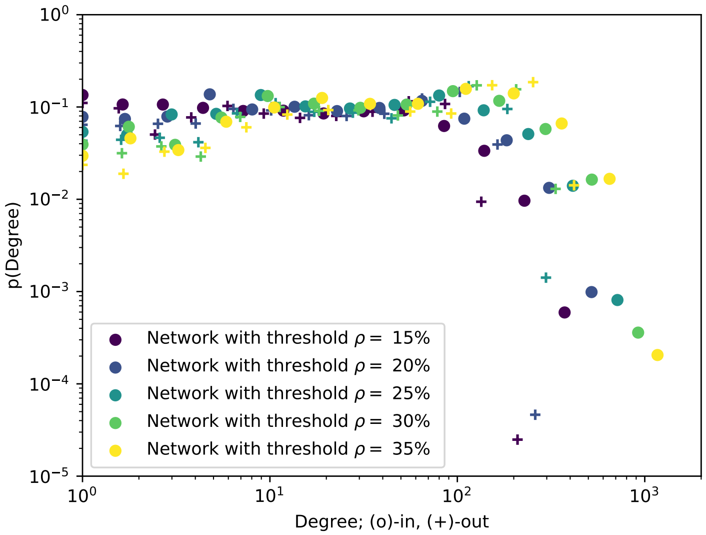

Figure S2 | All-to-all network distribution. In- and out-degree distribution for networks with differing thresholds ρ.

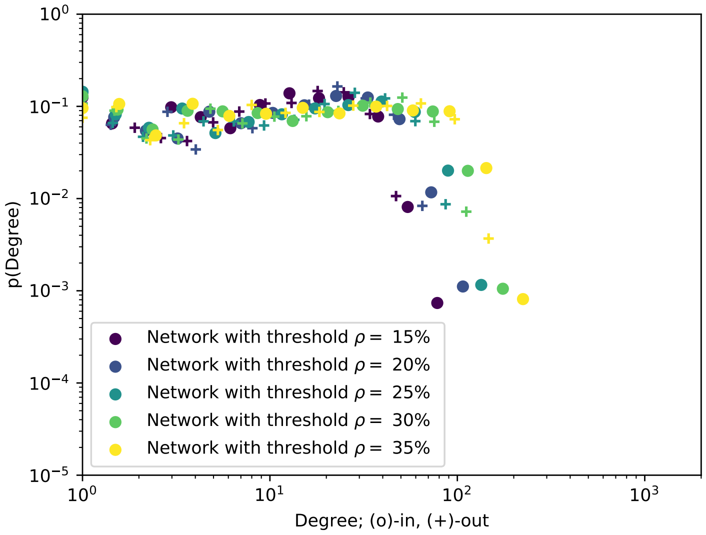

Figure S3 | Land-to-land network distribution. In- and out-degree distribution for networks with differing thresholds ρ.

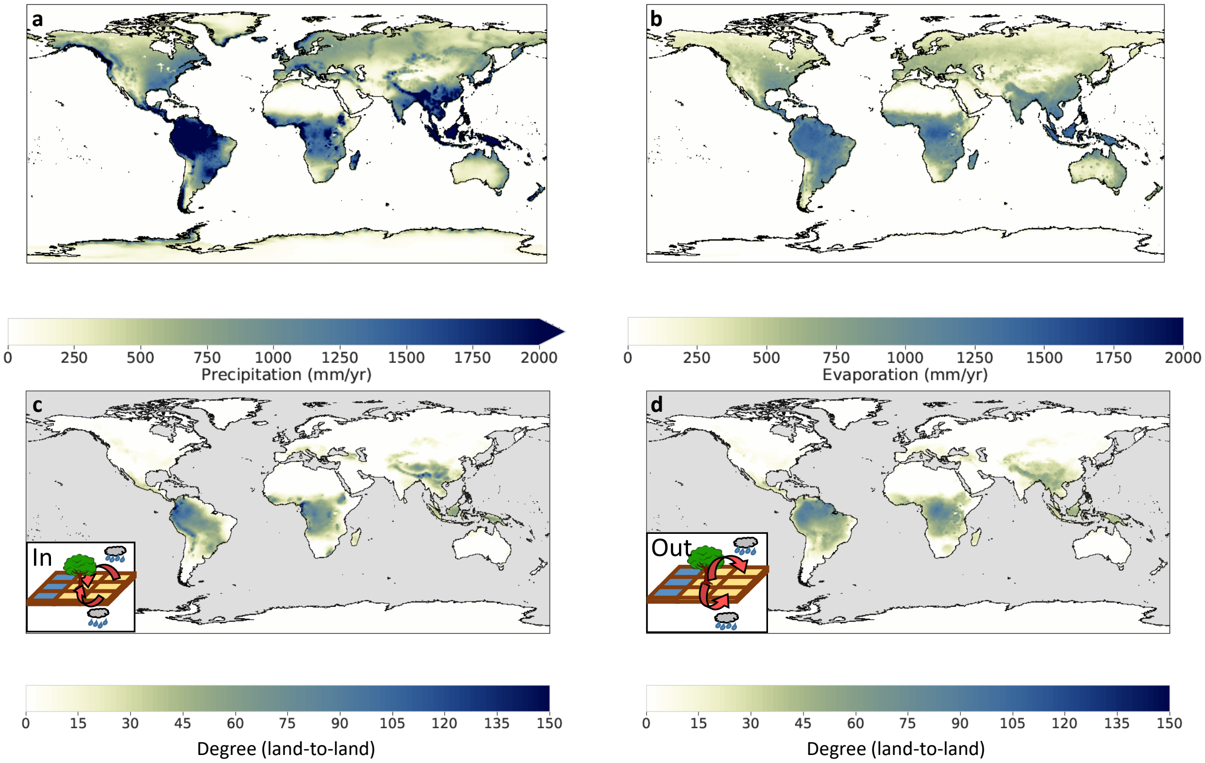

Figure S4 | ERA5 input data. **a,** ERA5 precipitation over land and **b,** ERA5 evaporation over land. **c,d,** In- and out-degree for land-to-land connections as shown in Fig. 1c,d of the main manuscript. The main moisture recycling hubs (super-receivers and super-distributors) are found by the precipitation and evaporation patters in **a, b** as well as by the network measures in **c, d**. However, the land-to-land network measures highlight the regions that start and end over land, meaning that the land-to-land connections are emphasized within the larger moisture recycling hubs.

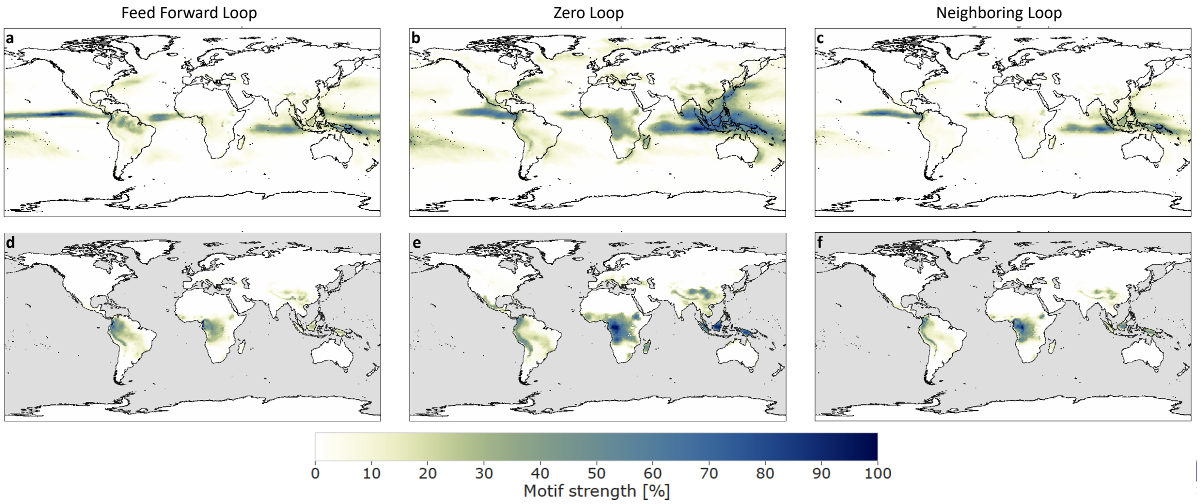

Figure S5 | Motif strengths per node. a-c, Motif strength per node for the all-to-all network, and **d-f,** for the land-to-land network. The oceanic regions are grayed out in the land-to-land network. **a, d,** Feed forward loop, **b, e,** Zero loop, **c, f,** Neighboring loop.

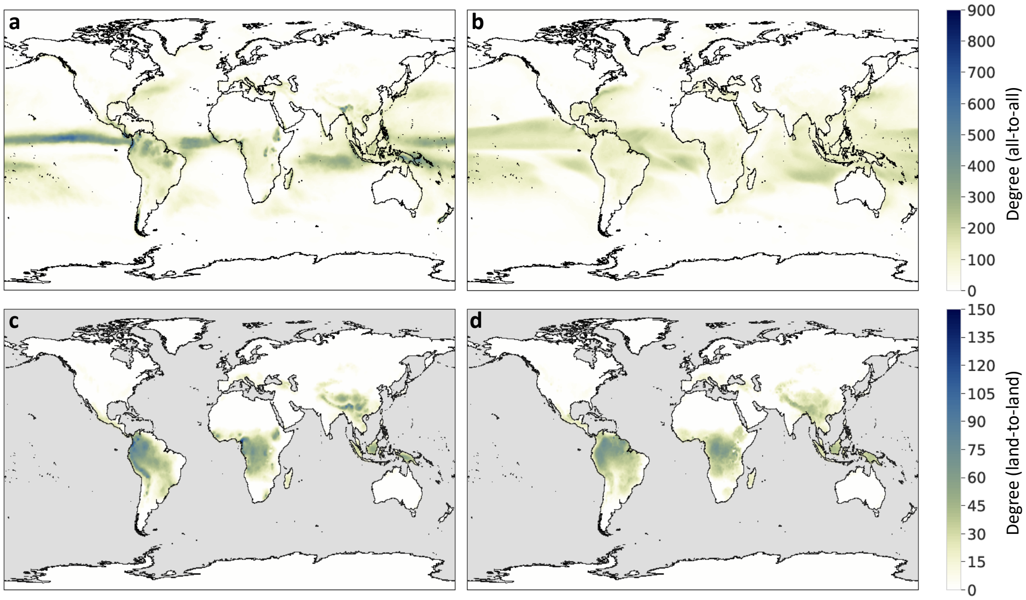

Figure S6 | Degree for threshold $\rho = 20\%$ **of all moisture. a,** In-degree for the all-to-all network. **b,** Out-degree for the all-to-all network. **c,** In-degree for the land-to-land network. **d,** Out-degree for the land-to-land network.

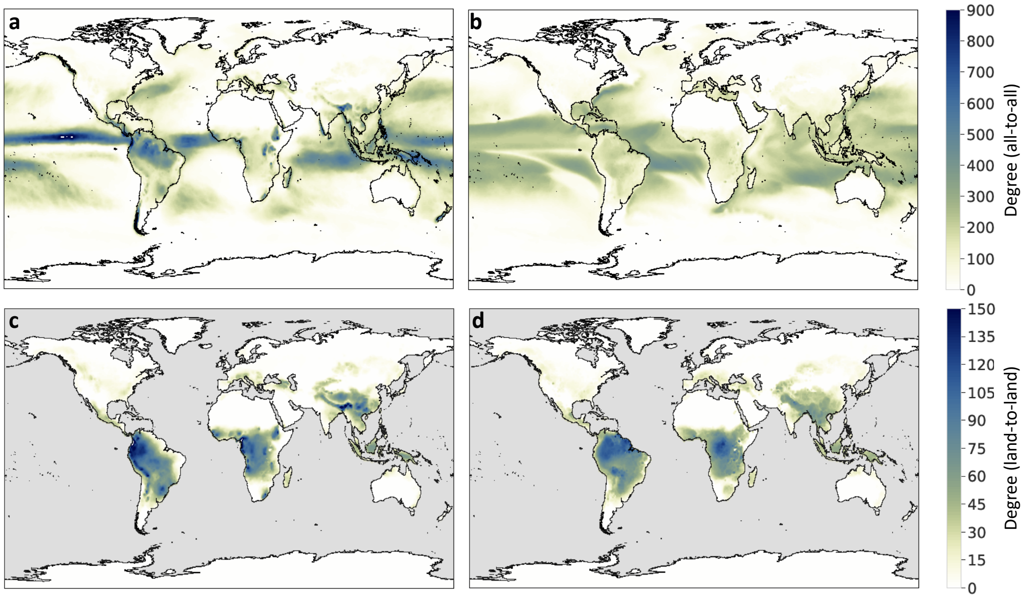

Figure S7 | Degree for threshold $\rho = 30\%$ of all moisture. **a,** In-degree for the all-to-all network. **b,** Out-degree for the all-to-all network. **c,** In-degree for the land-to-land network. **d,** Out-degree for the land-to-land network.

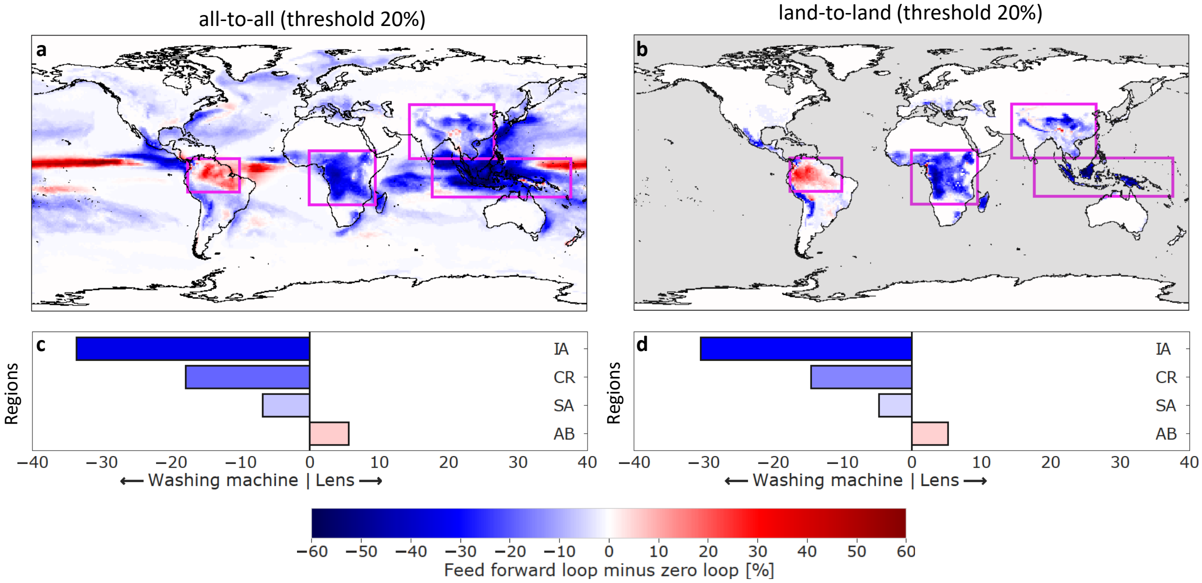

Figure S8 | FFL-ZL for $\rho = 20\%$. Feed forward loop minus zero loop occurrence for $\rho = 20\%$ of all moisture for **a,** the all-to-all network and **b,** the land-to-land network. The corresponding spatially aggregated averages are shown in panels **c** and **d**. Compare panels to Fig. 2a-d of the main manuscript.

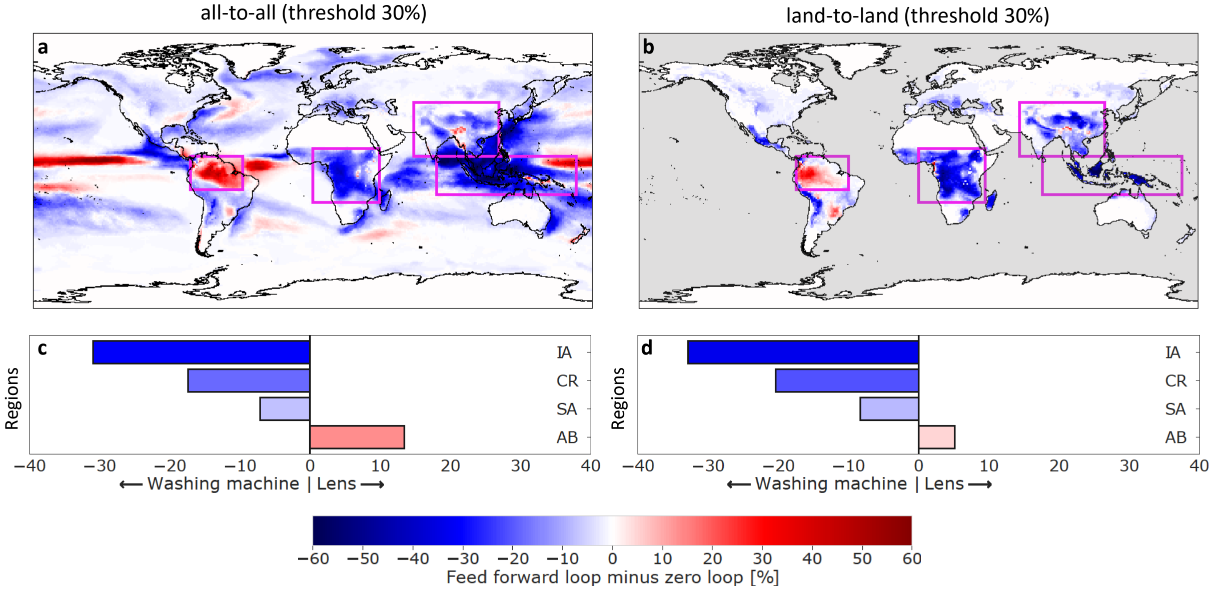

Figure S9 | FFL-ZL for ρ = 30%. Feed forward loop minus zero loop occurrence for threshold ρ = 30% of all moisture for **a,** the all-to-all network and **b,** the land-to-land network. The corresponding spatially aggregated averages are shown in panels **c** and **d**. Compare panels to Fig. 2a-d of the main manuscript.

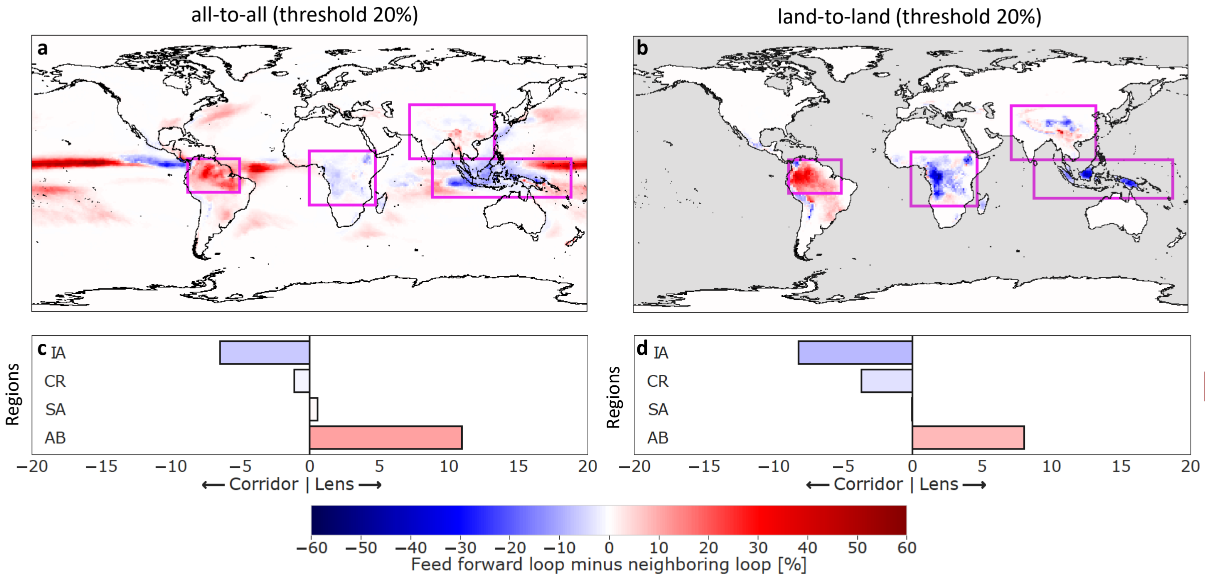

Figure S10 | FFL-NBr for ρ = 20%. Feed forward loop minus neighboring loop occurrence for threshold ρ = 20% of all moisture for **a,** the all-to-all network and **b,** the land-to-land network. The corresponding spatially aggregated averages are shown in panels **c** and **d**. Compare panels to Fig. 2e-h of the main manuscript.

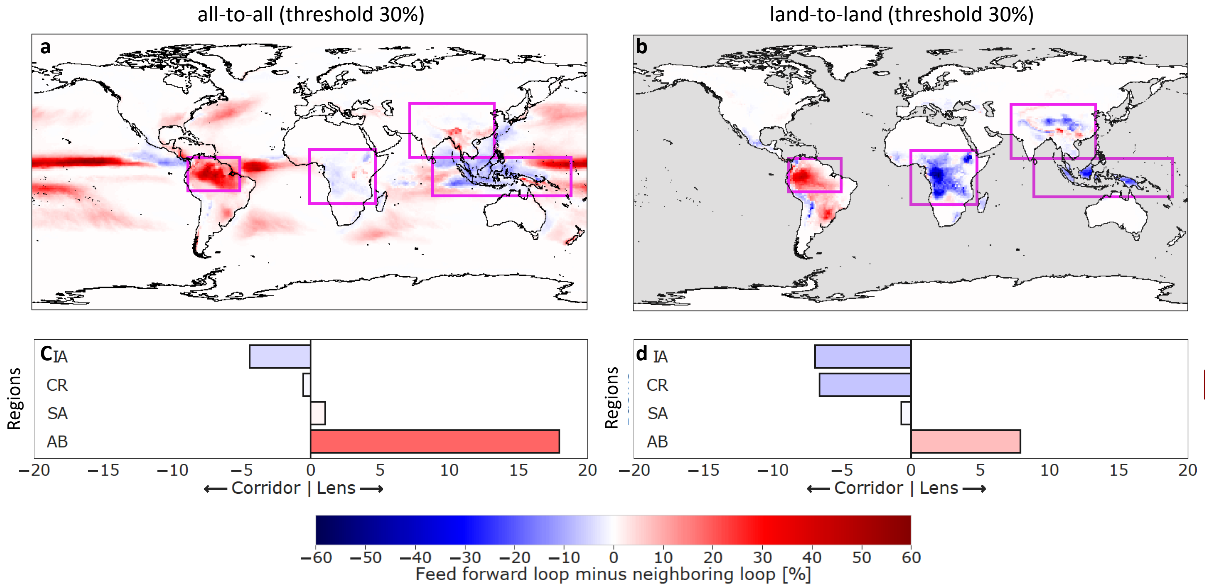

Figure S11 | FFL-NBr for $\rho = 30\%$. Feed forward loop minus neighboring loop occurrence for threshold $\rho = 30\%$ of all moisture for **a,** the all-to-all network and **b,** the land-to-land network. The corresponding spatially aggregated averages are shown in panels **c** and **d**. Compare panels to Fig. 2e-h of the main manuscript.

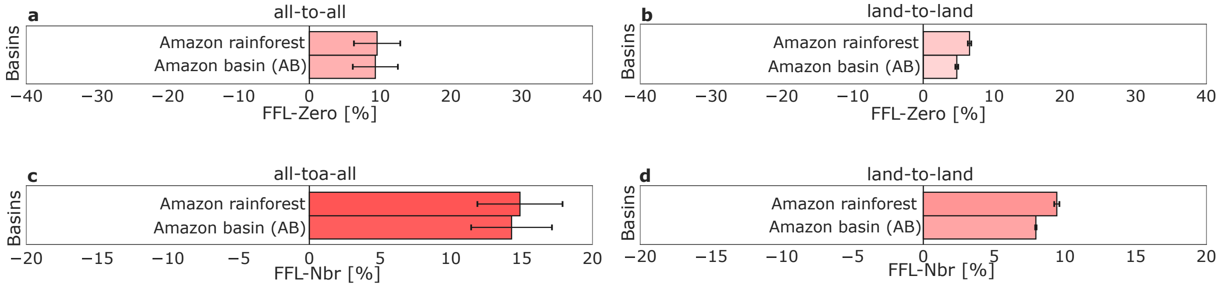

Figure S12 | Comparison between rectangle in South America and Amazon forest cover. Comparison between the computations in the main manuscript (Amazon basin, lower horizontal bar, see **c, d**) and the Amazon rainforest covered region (upper horizontal bar, see **a, b**). The four scenarios are the same as in Fig. 2 panels (c,d and g,h) of the main manuscript.

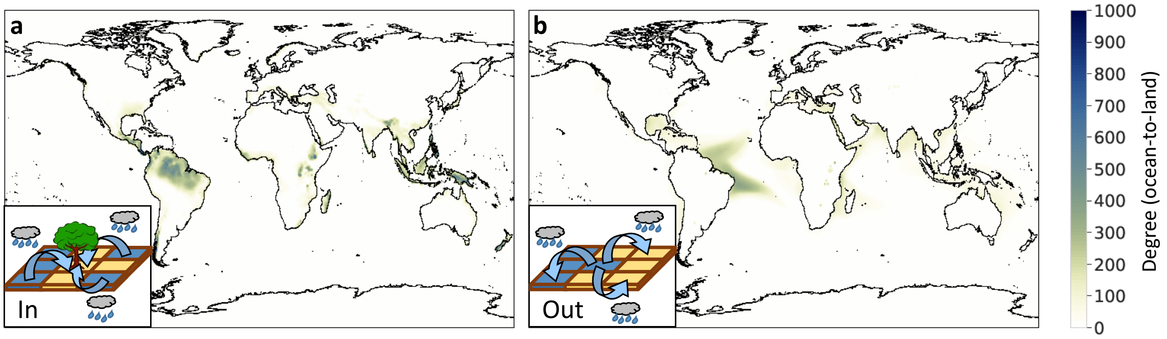

Figure S13 | Ocean-to-land network. **a,** In-degree, and **b,** out-degree as shown in the insets.

Ocean to land connectivity class

To study how different connectivity classes contribute to the overall linkage pattern and how they contribute to the presence of motifs in the focus regions, we analyzed the global all-to-all network together with the land-to-land network. Here, we additionally illustrate how the ocean-to-land network provides insights on where water masses contribute to moisture influx over land masses. By definition, only in-degree over land and out-degree over water exhibit nonzero values in Fig. S13.

The most prominent region in Fig. S13a (in-degree) is the northeastern part of the Amazon basin. Analyzing the associated pattern over the Atlantic in Fig. 1a reveals that the two branches north and south of the inter-tropical convergence zone serve as the moisture source. Similar effects of oceanic moisture influx but with the smaller spatial extent and lower in- and out-degree are visible in southeast Asia and the Indonesian archipelago. Furthermore, Fig. S13 also demonstrates how the large lakes in continental Africa distribute moisture around them: whereas small spots of high out-degree spatially coincide with the location of the lakes, larger regions of moderate in-degree in the surrounding regions confirm their regional importance for moisture distribution.

Connectivity classes

In the data set, different classes of connections are included. Specifically, there are connections from ocean to ocean, from ocean to land, from land to ocean and from land to land. Naturally, we can consider all of them, but also filter out specific link classes for addressing certain research questions. In this work, we are especially interested in the general organization of moisture transport globally in an "all to all" manner as well as in the moisture transport from land to land, i.e. identifying locations, where land is directly depending on moisture from other locations over land. That is we feature those

two classes (all-to-all and land-to-land) in the main manuscript, and discuss ocean-to-land moisture characteristics in the supplement (see above).

Motifs in randomized networks

Motifs do not appear similarly often in networks generally, but their occurrence heavily depends on the network structure and type of the network itself[2,3]. This is not only due to their different topology, but especially caused by the differing number of nodes included. Hence, there are multiple orders of magnitude more FFLs than ZLs in the moisture recycling network. This impedes a direct comparison of the motifs and their relative frequency, which is needed for identifying the dominating motif in the respective regions. As already stated, we, therefore, normalize the motif counts by the respective maximum to obtain the motif strength.

To conclude that a motif is *dominating*, even if it is actually less present than another, it is additionally needed to confirm that the occurrence is higher than expected and especially higher than we would expect the other motif to be present. One way to approach this problem is setting up surrogate networks and study the ratio between different motifs. Such approaches have been used earlier in network studies and are an established approach[4,5,6]. An appropriate surrogate network needs to, first, fulfill the same degree distribution, and, second, especially needs to preserve the link density (number of links in the network). Hence, we have used a directed configuration model[7] which is feeded by the adjacency matrix of the original network. Thus, it does not only fulfill the stated conditions, but also preserves the per-node in- and out-degree. The only difference is that the random directed configuration network (hereafter called random network) is constructed via rewiring using a random scheme. In the following, we analyze the results based on the all-to-all network and the land-to-land network.

Fig. S13 features the results for the all-to-all network. The left column (a,c,e) shows the values of the original network, whereas the right column (b,d,f) shows the values of the configuration-model-based random network. As expected[2,3], the numbers of the network motifs in the random network are two orders of magnitudes below the ones in the original network (Fig. S13a,c,e vs Fig. S13b,c,f). This confirms that the original network is *not* random and network motifs are a substantial part of the functionality of the real-world network.

In contrast to this systematic difference between the two networks, the ratio between the numbers of the motifs remains very similar. Specifically, the number of ZLs is three orders of magnitude below the number of FFLs (compare Fig. S13 panels a,c with b,d). Also the number of NBrs and FFLs are of the same order of magnitude for both the random and the original networks (compare Fig. S13 panels a,e with b,f). Moreover, and

even more importantly, the distributions of the counts closely agree when rescaled to the respective maxima (Fig. S13a,c,e vs Fig. S13b,c,f). In other words, the rescaled numbers of motifs (i.e. the motif strengths) in the original network follow a similar distribution as for the configuration model network, and the rescaling values differ by the same order of magnitude for both networks. Therefore, the differences between the motif strengths are meaningful, as they reveal to which extent and at which nodes certain motifs dominate relative to each other. As a consequence, Fig. S13f and Fig. S13h reveal a substantial difference between the random and the original moisture recycling network: the differences in the motif strengths are broader distributed at both tails for the FFL-ZL difference (feed forward loop minus zero loop) and appear shifted for the FFL-NBr difference (feed forward loop minus neighboring loop). These differences are direct consequences of the topology of the focus regions. In particular, the higher probability to find more positive values for the FFL-ZL difference than for the FFL-NBr difference are caused by the anomalies in the Amazon basin (AB).

Evaluating the land-to-land network and the according random network based on the directed configuration model further confirm our observations (see Fig. S15). All conclusions can be adapted straightforwardly: we observe the same ratios between FFLs, ZLs and NBrs (where the difference between ZLs and NBrs and FFLs are only two orders of magnitude), and the rescaled distributions roughly agree with each other. The difference between the number of motifs in the moisture recycling network and the random network is smaller for land-to-land than for all-to-all (compare Fig. S15g,h with Fig. S13g,h). This is probably caused by the absence of ocean to land connections in the land-to-land network of Fig. S15.

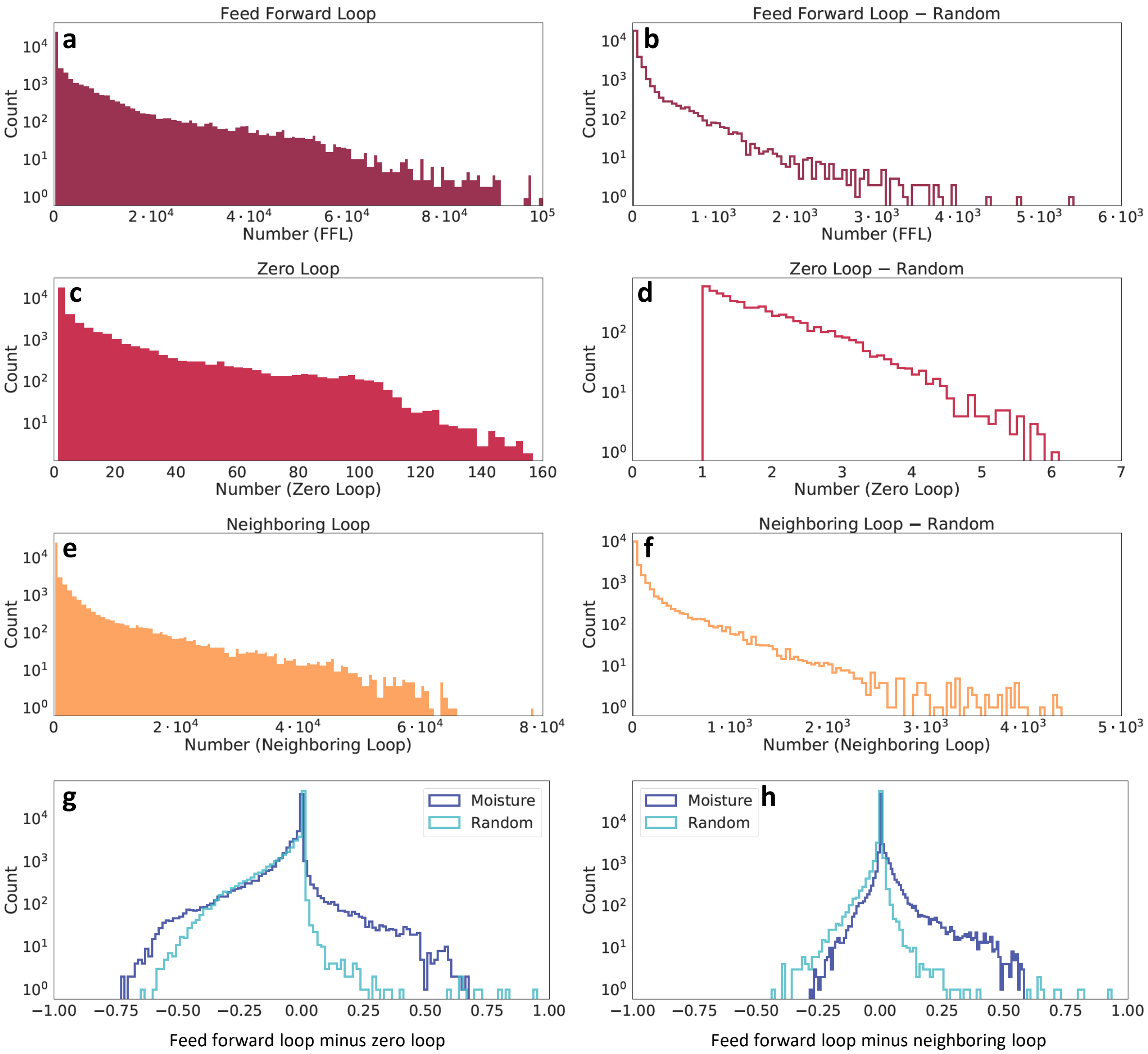

Figure S14 | Motif distribution in the all-to-all moisture recycling network. a, c, e, Distribution of motifs in the all-to-all moisture recycling network, as well as in **b, d, f,** the randomly generated network using a directed configuration model, which leaves the in- and out-degree sequences of the actual moisture recycling network constant and reshuffles the interconnections between different nodes. **g,** Distribution of normalized FFLs minus normalized number of zero loops. **h,** Distribution of normalized FFLs minus normalized number of neighboring loops. Here, $\rho = 25\%$ was used.

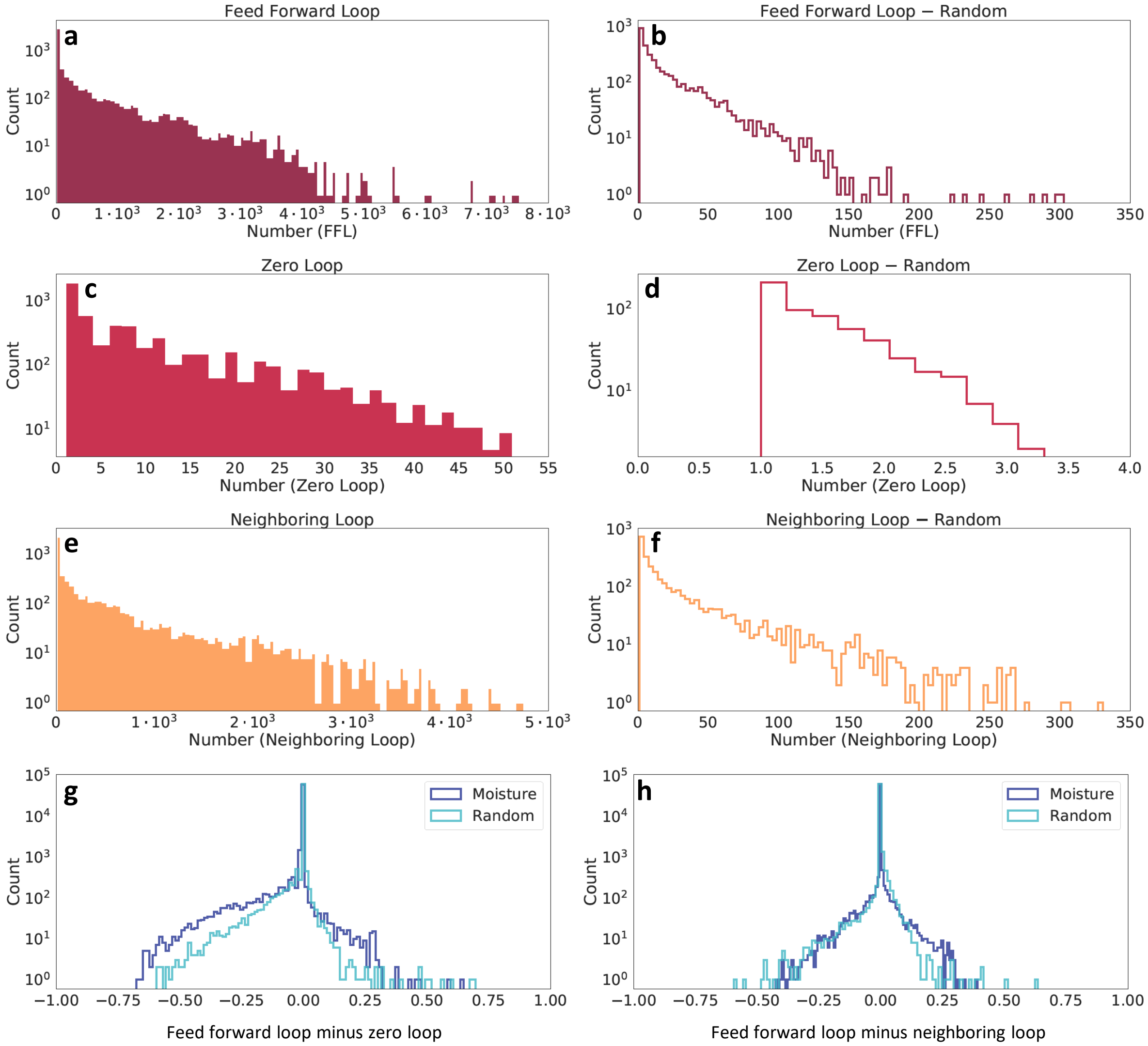

Figure S15 | Motif distribution in the land-to-land moisture recycling network. Same as Fig. S13, but for land-to-land connections only for $\rho = 25\%$.

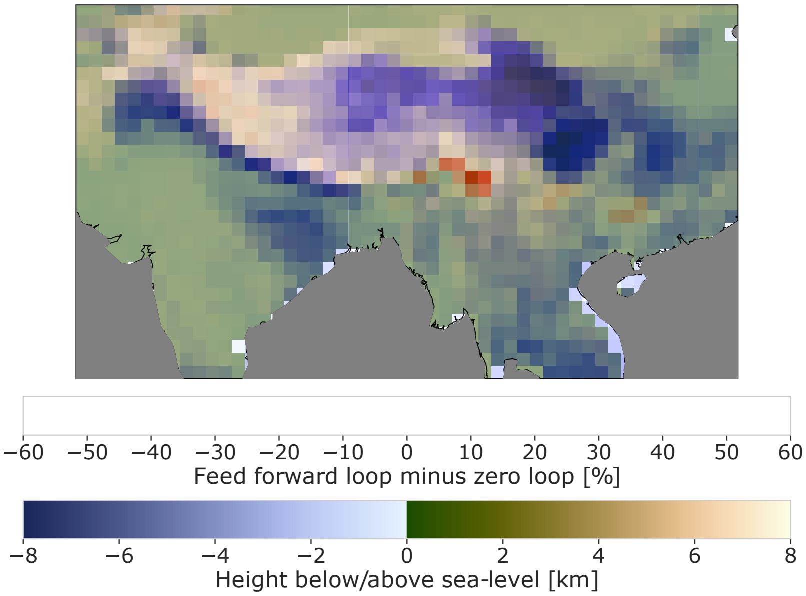

Figure S16 | The role of ZLs for South Asia. Topographic height elevation map overlaid with the normalized motif map (compare to global maps in Fig. 2a,b). The eastern and southwestern part of the Himalaya mountains are dominated by ZLs. This suggests that regions close to large height elevations are prone to possess many ZLs in comparison to FFLs.

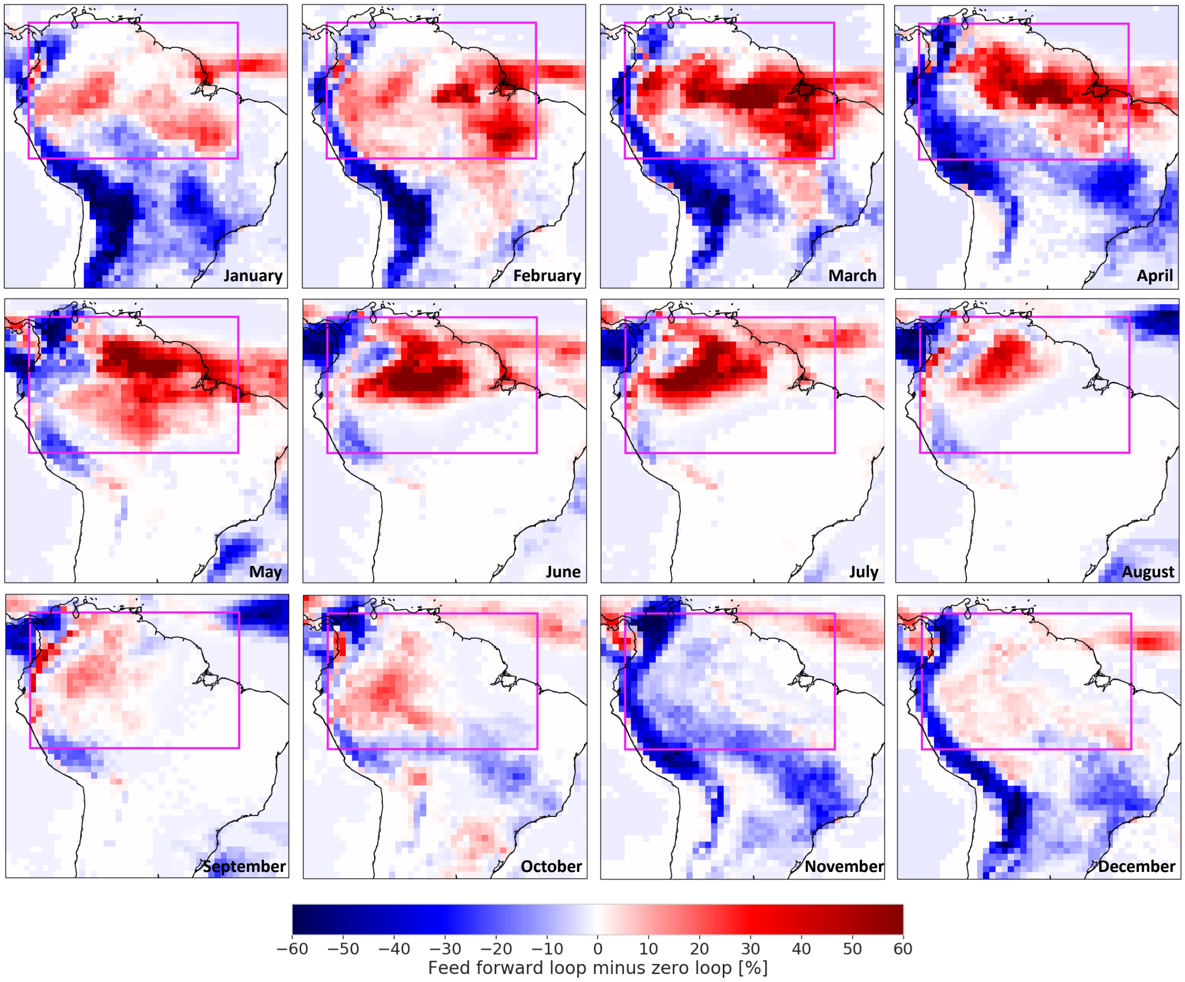

Figure S17 | Monthly characterization of the Amazon basin. FFL and ZL strength throughout the year for the all-to-all network for the Amazon basin (AB, see rectangle) and the larger South American region (compare to Fig. 2a in the manuscript).

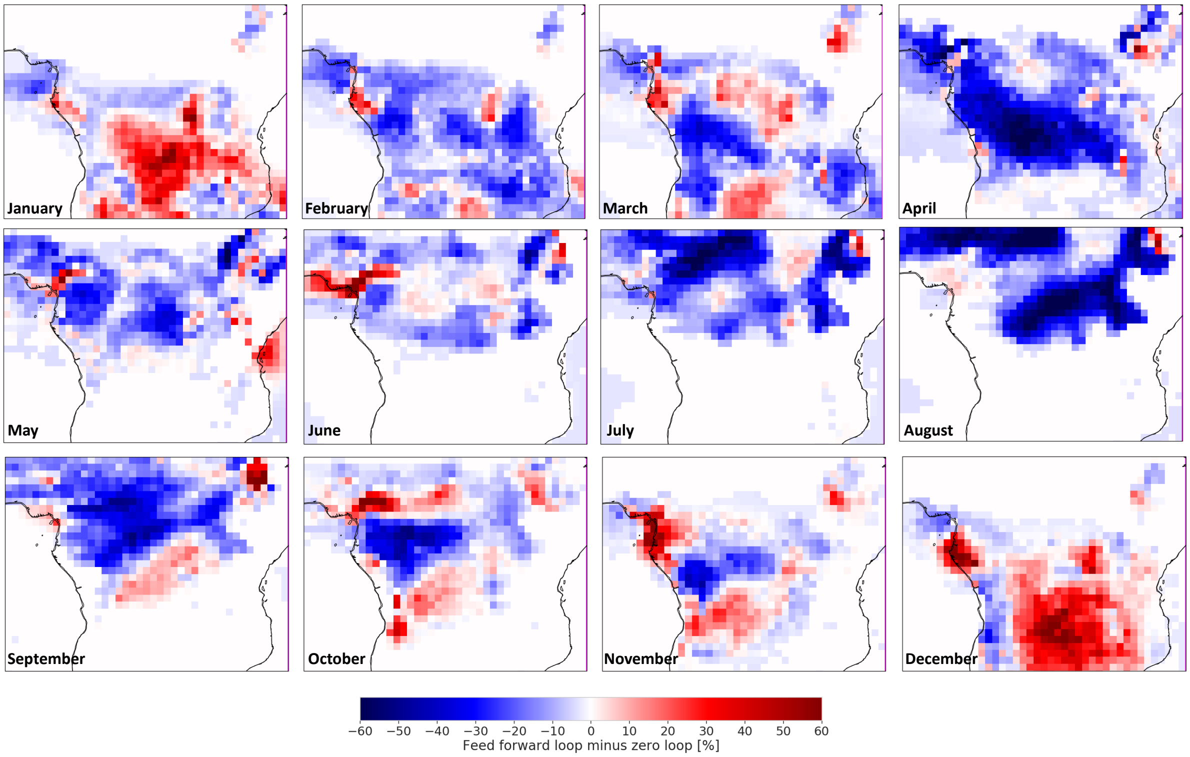

Figure S18 | Monthly characterization of the Congo rainforest. FFL and ZL strength throughout the year for the all-to-all network for the Congo rainforest (CR, compare to Fig. 2a in the manuscript).

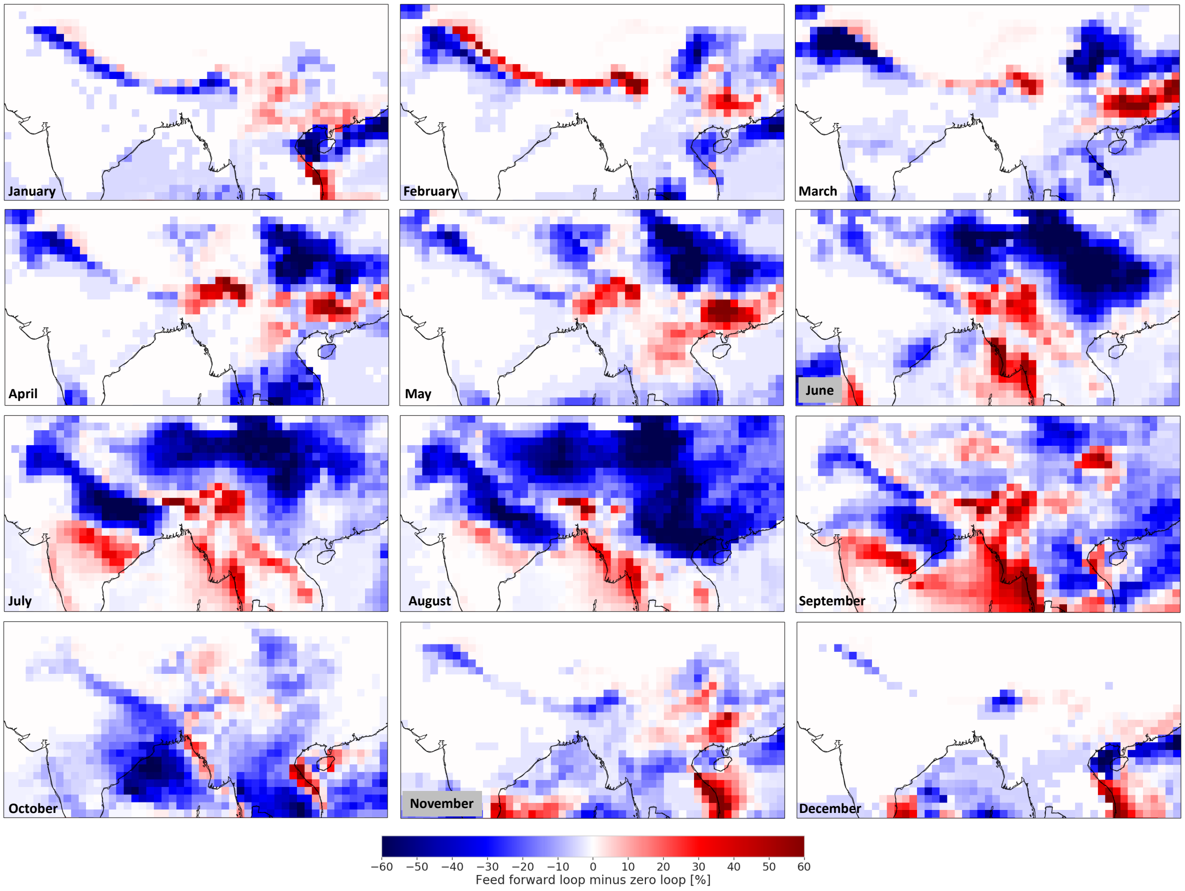

Figure S19 | Monthly characterization of South Asia. FFL and ZL strength throughout the year for the all-to-all network for South Asia (SA, compare to Fig. 2a in the manuscript).

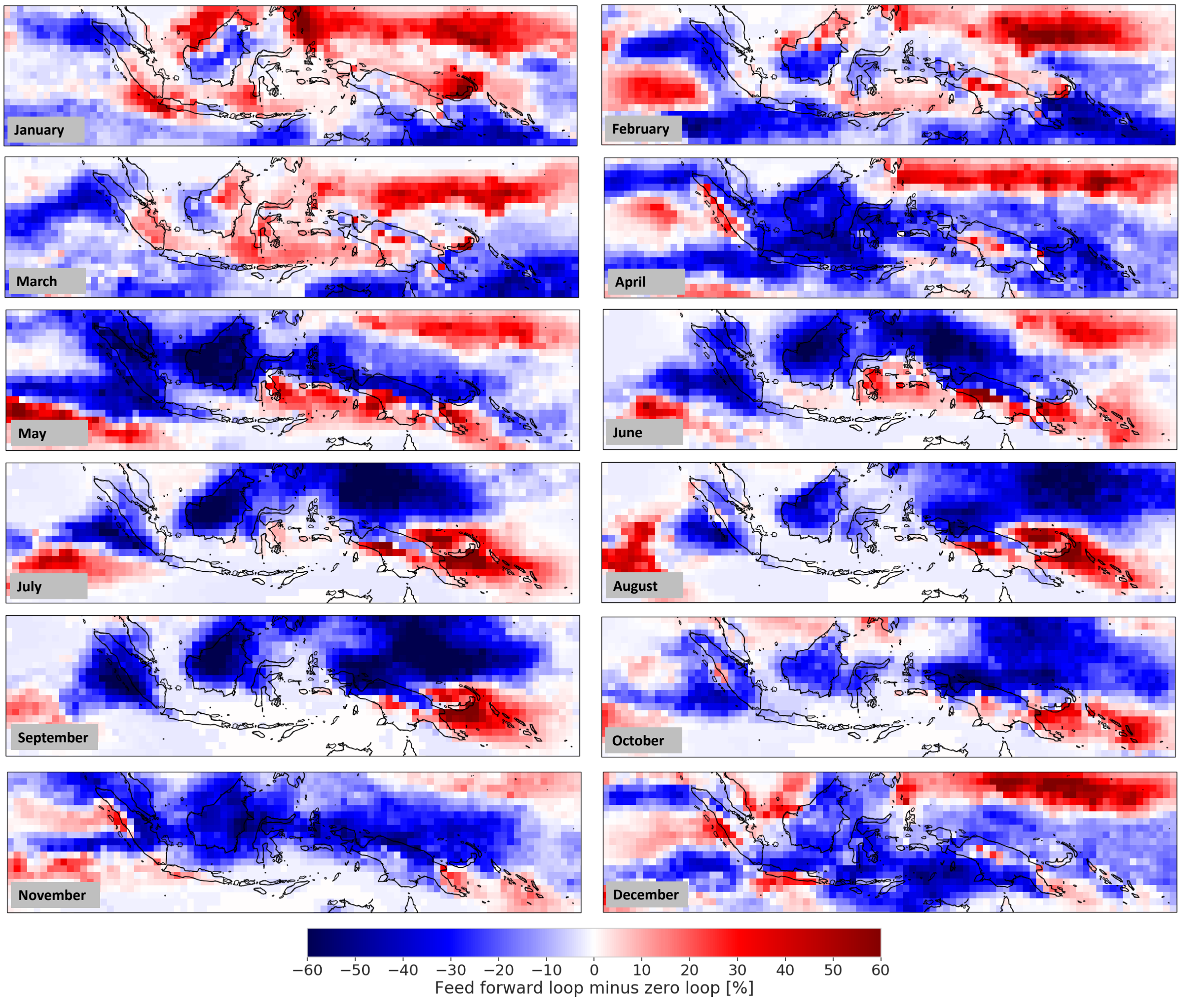

Figure S20 | Monthly characterization of the Indonesian Archipelago. FFL and ZL strength throughout the year for the all-to-all network for the Indonesian Archipelago (IA, compare to Fig. 2a in the manuscript).

Supplementary references

1. Barabási, A.-L. & Pósfai, M. *Network Science* (Cambridge University Press, 2016).

2. Wunderling, N. *et al.* How motifs condition critical thresholds for tipping cascades in complex networks: Linking micro-to macro-scales. *Chaos: An Interdisciplinary Journal of Nonlinear Science* **30**, 043129 (2020).

3. Milo, R. *et al.* Network motifs: simple building blocks of complex networks. *Science* **298**, 824–827 (2002).

4. Wiedermann, M., Donges, J. F., Kurths, J. & Donner, R. V. Spatial network surrogates for disentangling complex system structure from spatial embedding of nodes. *Phys. Rev. E* **042308**, 1–13 (2016).

5. Lekscha, J. & Donner, R. V. Phase space reconstruction for non-uniformly sampled noisy time series. *Chaos: An Interdisciplinary Journal of Nonlinear Science* **28**, 085702 (2018).

6. Wang, S., Liu, J. & Jin, Y. Surrogate-assisted robust optimization of large-scale networks based on graph embedding. *IEEE Transactions on Evolutionary Computation* **24**, 735–749 (2019).

7. Newman, M. E. The structure and function of complex networks. *SIAM Review* **45**, 167–256 (2003).
